# Supplementary material for: Data augmentation of time-series data in human movement biomechanics: A scoping review
Source: PLoS One. 2025 Jul 1;20(7):e0327038. doi: 10.1371/journal.pone.0327038 (PMC12212866; doi:10.1371/journal.pone.0327038)
Supplement: S1 Table — All publications that were included in the final selection of this review. (PDF) [file pone.0327038.s001.pdf]

S1 Table.

Included Publications. All publications that were included in the final selection of this review.

	Title	Author
[17]	Optimal Control Simulation Predicts Effects of Midsole Materials on Energy Cost of Running	Eva Dorschky et al.
[4]	CNN-Based Estimation of Sagittal Plane Walking and Running Biomechanics From Measured and Simulated Inertial Sensor Data	Eva Dorschky et al.
[22]	Insole-Based Estimation of Vertical Ground Reaction Force Using One-Step Learning With Probabilistic Regression and Data Augmentation	Roy Eguchi et al.
[10]	Acceleration Magnitude at Impact Following Loss of Balance Can Be Estimated Using Deep Learning Model	Tae Hyong Kim et al.
[23]	Spatiotemporal and Kinematic Characteristics Augmentation Using Dual-GAN for Ankle Instability Detection	Xin Liu et al.
[24]	Generative Deep Learning Applied to Biomechanics: A New Data Augmentation Technique for Motion Capture Dataets	Metin Bicer et al.
[26]	Prediction of Lower Limb Joint Angles and Moments during Gait Using Artificial Neural Networks	Marion Mundt et al.
[27]	IMU-to-Segment Assignment and Orientation Alignment for the Lower Body Using Deep Learning	Tobias Zimmermann et al.
[28]	Estimation of Gait Mechanics Based on Simulated and Measured IMU Data Using an Artificial Neural Network	Marion Mundt et al.
[31]	Subject-Independent, Biological Hip Moment Estimation During Multimodal Overground Ambulation Using Deep Learning	Dean D. Molinaro et al.
[32]	Low-Rank Representation of Head Impact Kinematics: A Data-Driven Emulator	Patricio Arruè et al.

	Title	Author
**[33]**	Estimation of Kinematics from Inertial Measurement Units Using a Combined Deep Learning and Optimization Framework	Eric Rapp et al.
**[34]**	The Use of Synthetic IMU Signals in the Training of Deep Learning Models Significantly Improves the Accuracy of Joint Kinematic Predictions	Mohsen Sharifi Renani et al.
**[12]**	Convolutional and Recurrent Neural Network for Human Activity Recognition: Application on American Sign Language	Vincent Hernandez et al.
**[35]**	Data Augmentation to Address Various Rotation Errors of Wearable Sensors for Robust Pre-impact Fall Detection	Xiaoqun Yu et al.
**[29]**	A Probability Fusion Approach for Foot Placement Prediction in Complex Terrains	Jingfeng Xiong et al.
**[36]**	From Mocap Data to Inertial Data Through a Biomechanical Model to Classify Countermeasure Exercises Performed on ISS	Martina Ravizza et al.
**[30]**	Estimation of Electrical Muscle Activity during Gait Using Inertial Measurement Units with Convolution Attention Neural Network and Small-Scale Dataset	Wenqui Liang et al.
**[37]**	Generative Data Augmentation of Human Biomechanics	Halldór Kárason et al.
**[11]**	Synthetic IMU Datasets and Protocols Can Simplify Fall Detection Experiments and Optimize Sensor Configuration	Jie Tang et al.
**[38]**	Strategies to Optimize Machine Learning Classification Performance When Using Biomechanical Features	Bernard Liew et al.
