# Supplementary material for: Data augmentation of time-series data in human movement biomechanics: A scoping review
Source: PLoS One. 2025 Jul 1;20(7):e0327038. doi: 10.1371/journal.pone.0327038 (PMC12212866; doi:10.1371/journal.pone.0327038)
Supplement: S2 Table — Data sources used in the individual publications, such as Motion Capture (MOCAP), inertial measurement units (IMUs), and electromyography (EMG). Additionally, the details of the data set created using the data sources is listed for each publication. No asterisk (*) denoted publications that used only their custom data set, acquired within the scope of the corresponding publication. * marks all publications that used previously recorded or public data sets. ** marks publications using a combination of own data and previously recorded or public data. Numbers with exponent c were corrected based on the reference and participant details provided. (PDF) [file pone.0327038.s002.pdf]

S2 Table.

Data Sources and Data Set Details. Data sources used in the individual publications, such as Motion Capture (MOCAP), inertial measurement units (IMUs), and electromyography (EMG). Additionally, the details of the data set created using the data sources is listed for each publication. No asterisk (*) denoted publications that used only their custom data set, acquired within the scope of the corresponding publication. * marks all publications that used previously recorded or public data sets. ** marks publications using a combination of own data and previously recorded or public data. Numbers with exponent c were corrected based on the reference and participant details provided.

	**Data Sources**	**Data Set Details**
[**17**]*	MOCAP Instrumented Treadmill	Used a public data set from [43] containing 28 participants running at different speeds.
[**26**]*	MOCAP Force Plate	Contains data from multiple studies conducted at the German Sport University in Cologne and included trials of participants walking at self-selected speeds. Some of the participants were knee arthroplasty patients.
[**27**]**	MOCAP IMUs (pelvis, upper and lower leg, foot)	Used own recordings as well as data from publicly available MOCAP data sets. Data set A: Simulated IMU data from 42 participants performing different walking styles [44]. B and C: Collected real IMU data of four male participants walking back and forth for one minute and 28 participants (13m, 15f) walking six minutes in an eight shape. The data sets B and C are not available anymore using the reference given in their publication.
[**4**]*	MOCAP Force Plate IMUs (lower back, right thigh, right shank, right foot	The authors used data previously collected in [45] including 595 walking and running cycles in total, performed by 10 male participants walking and running at six different speeds with 10 trials each.

	Data Sources	Data Set Details
[**28**]**	MOCAP Force Plate	The validation set recorded in this publication contained 30 healthy participants (18m, 12f) walking 10 level trials at five different speeds on a 5m walkway. The data of seven participants was excluded due to connection issues and data loss. Additionally, a previously collected data set containing 93 participants (55m, 38f) was used for training [46].
[**31**]	MOCAP Force Plate IMU (trunk, right thigh	16 participants (10m, 6f) completed 10 circles of level ground walking in three different speeds each. Additionally, they completed 10 trials of ramp ascent/descent and stair ascent/descent for different slopes and stair height. IMU data was discarded due to data dropout and was replaced by synthetic data
[**32**]*	Mouthgard designed in [47]	A previous collected data set of 573 head impact kinematic measurement during contact sports was used, containing 6 degrees of freedom kinematics for each impact [47,48].
[**33**]*	MOCAP	Participants performed 60-seconds walking and running tasks with self-selected speeds on a treadmill. While some participants were pain-free, others suffered a lower extremity running-related injury. Walking trials from 420 participants (203m, 217f) and running trials from 580 (292m, 288f) were used for the studies.
[**34**]	MOCAP Force Plate IMUs (pelvis, left thigh, left shank, left foot)	31^c participants (12m, 19f), where 13 of them had osteoarthritis, performed 15 trails of 5m walking tasks in three different speeds.

	Data Sources	Data Set Details
[12]	MOCAP	25 male novices in sign language. Each sign (numbers 0 to 10 and 49 words) was taught to them before measurement. In total, 16890 labeled signs were recorded with 60 features representing the kinematics of the right and left sides.
[22]	Pressure Insole Force Plate	Six healthy participants (5m, 1f) walked with a self-selected pace with insole pressure sensors over force plates. They estimated the vGRF based on insole pressure.
[10]	IMU (left side of pelvis)	24 participants (14m, 10f) mimic falls occurring among elders in 5 fall directions. In total 1278 falls were recorded.
[35]	IMU (lower back)	The data set used for data augmentation was recorded with 30 participants (15m, 15f) without rotation errors. The validation set was recorded with 12 new participants (6m, 6f) with sensor rotation errors. Each subject performed 21 types of daily activities and 15 falls. All tasks were repeated 5 times, except for static motions that were only repeated once.
[29]	Depth Camera (Waist) IMUs (foot, waist)	12 participants walked in different settings for 3 min (level ground, ramp ascent/descent) and 10 min (stairs ascent/descent), respectively with three different walking speeds.
[23]	MOCAP	30 gait cycles were collected of three male patients diagnosed with CAI walking and 211 normal gait cycles of 10 male control participants with no injuries walking.
[24]	MOCAP	Collection of 55 gait cycles across all eight, healthy, participants (4m, 4f) on a horizontal walkway.

	Data Sources	Data Set Details
[**36**]**	MOCAP<br>Force Plate	Two male athletes and six non astronaut participants (3m, 3f) performed a set of 4 repetitions of squads, wide stance squads and deadlift in correct execution as well as one set of each exercise with wrong execution. Additionally, previously collected experimental data at NASA Johnson Space Center in Houston was used.
[**38**]*	Force Plate	In the first data set [49], 2295 participants (1740 m, 555f) walked at a self-paced speed along 10m across two force plates either barefoot or wearing shoes. A total of 75732 bilateral trials were recorded. The second data set [50] contained pressure data of 31 participants (17 healthy and 14 with patellofemoral pain syndrome) performing three trials of CMJ using a self-determined depth. Only GRFs from one side were collected.
[**11**]**	Cameras (right and left front side)<br>IMUs (head, chest, waist, front of right thigh and shank, right ankle and right wrist)	They collected 16 fall and 16 non-fall actions, each performed three times, of 6 participants. 513 valid samples were collected. Additionally, a public data set [51] was used containing 20 fall and 16 non-fall actions performed by 14^c participants (7m, 7f) repeated between 5 and 6 times. Together 3133 fall samples were provided.

	Data Sources	Data Set Details
**[30]**	EMG (biceps femoris, gastrocnemius medialis, gastrocnemius lateralis) IMUs (shank, thigh)	Seven participants (4m, 3f) walked on a treadmill in a self-selected speed, slow (0.8 times the selected speed) and fast (1.2 times the selected speed). Thereby, data from the sensors was collected for 3 trials for each participant over 40 seconds each. In total, 215 slow, 241 normal, and 258 fast gait cycles were collected.
**[37]***	EMG Force Plate IMUs (trunk, thigh, shank, foot) Goniomether (Hip, knee, ankle) MOCAP	The authors used a public data set [52] containing walking data from 22 healthy participants (13m, 9f). Hereby, the participants walked in different conditions, including walking on a treadmill, ground level, stairs, ramps at different speeds, stair height, and ramp inclination. A total of 37687 gait cycles is provided.
