# Supplementary material for: Data augmentation of time-series data in human movement biomechanics: A scoping review
Source: PLoS One. 2025 Jul 1;20(7):e0327038. doi: 10.1371/journal.pone.0327038 (PMC12212866; doi:10.1371/journal.pone.0327038)
Supplement: S3 Table — Reported evaluation methods and results of data augmentation across publications. (PDF) [file pone.0327038.s003.pdf]

S3 Table.

Data Augmentation Results. Reported evaluation methods and results of data augmentation across publications.

	**Evaluation**	**Results**
**[17]**	No evaluation of augmentation method.	
**[26]** *	Evaluation of the performance of the downstream model trained on data sets with different numbers of augmented samples	They found that their simulated data corresponds to real data, however, it does take soft tissue into account. Additionally, data augmentation improved the prediction results of the models. However, they did not provide numbers for the improvements achieved by data augmentation.
**[27]**	Evaluation of synthetic IMU by comparing it to real IMU signals and simulated IMU signals obtained from IMUsim [53]. Comparison of model performance trained on augmented data and non-augmented data based on accuracy.	They found a synthetic gap between real and simulated IMU that could be due to clothing or soft tissue. They reported that recorded data alone is not sufficient, and also training on simulated data alone decreases the performance. However, adding a small amount of recorded IMU data increased the performance up to 92% accuracy and in summary reported that a combination of simulated and real data yielded promising results.

	Evaluation	Results
[4]	Comparison of model performance trained on augmented data and non-augmented data based on RMSE and Pearson Correlation Coefficient (PCC).	They showed that adding simulated data to the training decreases the RMSE of the joint angles by up to 31%. PCC improved from 0.96-0.99 to over 0.98 when simulated data is included. However, adding simulated data decreased the performance of hip joint estimates. They also found that simulated data was less noisy than measured data, which can also be related to soft tissue, as mentioned in [27]. It was also reported that overfitting on simulated data could be seen, especially for vertical ground reaction forces.
**[28]**	Comparison between measured and simulated data using the RMSE, correlation coefficient and accuracy. Comparison of model performance trained on augmented data and non-augmented data.	In their case, the simulated data was a good representation of the measured IMU data, with the pelvis sensor achieving the highest accuracy (0.95 ± 0.08). However, higher gait velocities showed larger derivations between simulated and measured data, which they also attributed to soft tissue movement. Worth mentioning is that they found, that prediction of kinematics increases with more data samples. Introducing additional noise led to better results than increasing the sample size alone.
**[31]**	No evaluation of augmentation method.	

	Evaluation	Results
[32]	Visually compared the distribution of the original data set with the distribution of a generated data set containing the same number of samples.	The synthetic data set had similar distribution as the original data set. Features in the created data set also have no significant differences to the original features, such as peak duration distribution.
[33]	Comparison of model performance trained on augmented data and non-augmented data based on the RMSE.	They show that models trained on augmented data showed a lower RMSE when tested on augmented data, however, not when tested on non-augmented data. Still, they reported that with a higher number of samples, the RMSE decreases.
[34]	Comparison of model performance trained on augmented data and non-augmented data based on the RMSE and correlation coefficient. Comparison of synthetic IMU signals and real IMU signals.	They report that when trained on simulated and measure data, there is a 54% reduction in RMSE and a 20% improvement of the correlation coefficient. The showed an average improvement of RMSE for joint angle predictions of 38% at the hip and 11% on the knee when the model was trained only using synthetic data and an improvement of 54% and 45% when trained on synthetic and measured data. Additionally, they report that the predictions for pelvis signals were worse than predictions from other body locations contradicting [28].
[12]	Comparison of model performance trained on augmented data and non-augmented data.	They reported a significant improvement of 3.8% accuracy when data augmentation was used.

	Evaluation	Results
[22]	Comparison of model performance trained on augmented data and non-augmented data based on accuracy, normalized RMSE and normalized error.	Simulating up to three virtual steps for augmenting the data set improved the performance of the models. However, generating more than four steps did not enhance the performance of the model.
[10]	Comparison of model performance trained on augmented data and non-augmented data based on the mean absolute percentage error (MAPE). Comparison of different augmentation methods and, additionally, the performance of all augmentation methods applied to one data set.	The average MAPE values were significantly ($p = 0.01$) decreased by 55.8% when data augmentation was used. They found no significant difference between the results of the different data augmentation techniques and the results when using all augmentation techniques.
[35]	Comparison of model performance trained on augmented data and non-augmented data.	They reported, that the two models trained on augmented data outperformed the model trained on non-augmented data with mean improvements of 6.11% and 6.5% when looking at the accuracy on the non-augmented validation set. With increasing improvement when the range of rotation error increased. There was no significant difference between the two different augmentation methods.

	Evaluation	Results
**[29]**	Comparison of model performance trained on augmented data and non-augmented data based on the RMSE and landing feasible area accuracy (LFAA) performance.	Data Augmentation improved the result by 10% RMSE and 0.74% LFAA. Stated that data augmentation enhances the prediction accuracy of foot placement.
**[23]**	Verification of synthetic IMU based on t-distributed stochastic neighbor embedding. Used different data sets to compare the model performance. Additionally, comparison of model performance trained on augmented data and non-augmented data.	They found that the simulated data followed the distribution of the real data. They reported that the number of training samples is crucial for the models' performance. When adding 100 and 200 synthetic data points, the outcome of the detection model was improved.
**[24]**	Comparison of model performance trained on augmented data and non-augmented data based on accuracy.	They reported that their data augmentation approach increased the accuracy of estimating joint kinematics by 23% compared to the model trained on original data only.
**[36]**	No evaluation of augmentation method.	

	Evaluation	Results
[38]	Comparison of model performance trained on augmented data and non-augmented data using multiclass Brier score, area under the curve, balanced accuracy and logarithmic loss	They reported different performances of the models but did not give a clear evaluation of the augmentation technique. It is reported that for data set A, the best model was trained on using a data set of 8 and 12 times the original size. For data set B, the best model was trained on 2 times the original size when looking at the brier score and 8 and 12 times the original size for the accuracy.
[11]	The authors illustrated the simulated and real IMU signals and compared the performance when using synthetic data and using experimental data for training.	For non-fall data, the signals of synthetic IMU and measured IMU were similar. For fall, however, the deviation was more significant, which can be due to discrepancy of simulated IMU positions on the model in the simulation environment and the actual IMU placement on subjects. The accuracy improved 4.49% when trained on simulated instead of experimental data. For the second data set, it is 0.58% worse than the accuracy achieved when trained on experimental data

	**Evaluation**	**Results**
**[30]**	Comparison of distribution between real and synthetic data using principal component analysis, correlation coefficient, and t-distributed stochastic neighbor embedding. Additionally, comparison of methods trained on data sets containing different amount of synthetic data (1.5 and 2 times training data size).	They found that the distribution of real and synthetic data highly overlap, indicating high similarity. Additionally, the PCC is increased with training size.
**[37]**	Compared the synthetic and real ground reaction forces and kinematic coordinate trajectories using statistical parameter mapping (SPM) two-tailed paired t-test ($\alpha = 0.05$). Additionally, t-distributed stochastic neighbor embedding was used to give a qualitative analysis.	It was found that the model is unable to generate synthetic data for unseen subjects. They discard their hypothesis that it is possible to train a generative adversarial network to generate realistic movement by only using anthropometric measures of the subjects, due to the time-series data being subject-dependent. Despite, the SPM had only few non-similarities.
